# Effect of a Plaque‐Identifying Toothpaste on Plaque Amount in 12–16‐Year‐Olds With Fixed Orthodontic Appliances: A Randomised, Double‐Blind, Controlled Clinical Trial

**DOI:** 10.1111/ocr.12904

**Published:** 2025-02-28

**Authors:** Sebastiaan P. van Doornik, Lisa van Kammen, Yijin Ren, David J. Manton, Anne Marie Kuijpers‐Jagtman

**Affiliations:** ^1^ Department of Orthodontics University of Groningen, University Medical Center Groningen Groningen the Netherlands; ^2^ Center for Dentistry and Oral Hygiene University of Groningen, University Medical Center Groningen Groningen the Netherlands; ^3^ Department of Paediatric Dentistry Academic Centre for Dentistry Amsterdam (ACTA), Vrije Universiteit Amsterdam and University of Amsterdam Amsterdam the Netherlands; ^4^ Department of Orthodontics and Dentofacial Orthopedics, School of Dental Medicine/Medical Faculty University of Bern Bern Switzerland; ^5^ Faculty of Dentistry Universitas Indonesia Jakarta Indonesia

**Keywords:** dental plaque, fixed buccal appliances, oral hygiene, orthodontic treatment, Orthodontics, Preventive Dentistry, quantitative light–induced fluorescence, toothpaste

## Abstract

**Objective:**

To evaluate the efficacy of a plaque‐identifying toothpaste in assisting with plaque removal in adolescents undergoing orthodontic treatment with fixed appliances.

**Methods:**

A randomised, double‐blind, active‐comparator trial was conducted at the Orthodontic Department, University Medical Center Department, University Medical Center Groningen, the Netherlands, from October 2021 to January 2022. Seventy seven healthy adolescents aged 12–16 years, undergoing buccal fixed appliance treatment for at least 3 months, were enrolled. Participants were stratified based on manual or electric brushing habits and randomised to receive either plaque‐identifying toothpaste or a non‐colouring control toothpaste. The primary outcome measured reduction in dental plaque using Quantitative Light‐Induced Fluorescence ∆R30 (QLF) images at baseline (T0) and after 4–7 weeks (T1). Secondary outcomes included self‐reported participant experiences.

**Results:**

No statistically significant difference in plaque reduction was found between the two groups (*F* = 0.211, *p* = 0.647). Subgroup analyses showed that participant‐related factors did not significantly influence plaque reduction. Participants using plaque‐identifying toothpaste reported a slightly stronger perception of plaque removal (*p* = 0.018) but encountered slightly more difficulty with the toothpaste colour (*p* = 0.028). Compliance was high, and no adverse effects were reported.

**Conclusion:**

Plaque‐identifying toothpaste did not lead to significant plaque reduction among adolescents with fixed orthodontic appliances.

## 1 Introduction

The dysbiotic microbiota of the dental biofilm pose a significant threat to oral health [1]. The frequent consumption of fermentable carbohydrates, particularly free sugars, creates an acidic and hypoxic environment within plaque, favouring the proliferation of cariogenic microorganisms [2]. Orthodontic treatment with fixed appliances exacerbates plaque accumulation due to the introduction of foreign materials and an increase in retention sites [3]. Fixed orthodontic appliances pose a challenge to mechanical plaque removal, thereby increasing the risk of caries during treatment, as evidenced by studies reporting a range of carious lesion prevalence from 33% before treatment to 68% after 12 months of treatment [4, 5, 6]. Effective plaque removal, primarily through mechanical means, is vital for reducing gingivitis and caries risk [7, 8]. While toothpaste, especially fluoride‐containing variants, enhances plaque removal and caries prevention [9], toothbrushing alone often falls short in adequately cleaning areas around orthodontic brackets [3]. Supplementation with interdental cleaning devices (dental floss and interdental brushes) improves plaque and gingivitis control [10].

With a growing number of individuals undergoing orthodontic treatment [4], enhancing oral hygiene practices becomes paramount, especially considering that adolescents often struggle to adhere to oral hygiene guidelines [11]. Numerous motivational strategies, including verbal, written and visual methods, have been researched, showing a significant positive effect on plaque and gingival bleeding scores among patients with fixed orthodontic appliances [12]. Making plaque visible could enhance awareness of plaque retention areas, thereby encouraging more effective cleaning [13, 14]. A promising method involves the use of plaque‐identifying agents, such as blue colour index (CI) 42090 and red CI 45410. These agents can be administered professionally as a ‘disclosing’ solution or as chewable tablets, or incorporated into toothpaste formulations [15]. Plaque‐identifying tablets and toothpastes alongside motivational conversations about oral health are reported to be effective in improving oral health in adults with fixed orthodontic appliances [15, 16, 17, 18].

Notably, no previous studies have explored the effect of a plaque‐identifying toothpaste on plaque reduction in adolescents with orthodontic fixed appliances. Unlike other plaque‐identifying agents, a plaque‐identifying toothpaste integrates into daily oral care routines with recent studies also exploring various types of plaque‐identifying toothpaste in (non‐) orthodontic patients [15, 16, 18]. It was hypothesised that a plaque‐identifying toothpaste can benefit these patients. Thus, this randomised, double‐blind, active‐comparator trial aimed to evaluate the reduction of plaque fluorescence of a plaque‐identifying toothpaste compared to a regular toothpaste in individuals undergoing orthodontic treatment with fixed appliances.

## 2 Subjects and Methods

### 2.1 Trial Design and Setting

This study, conducted at the Orthodontic Department of the University Medical Center University Medical Center Groningen (UMCG), the Netherlands, employed a randomised, double‐blind, active‐comparator trial methodology with a parallel design and equal allocation ratio based on brushing habits. The study protocol was registered in the ISRCTN registry (number ISRCTN53131830) and is accessible online at https://www.isrctn.com/ISRCTN53131830.

The Medical Ethics Institutional Review Board of the UMCG reviewed the research protocol and determined that the study did not meet the criteria of the Medical Research Involving Human Subject Act. Therefore, exempt approval was granted (WMO; registration number: METc 2021/516).

Study data were collected using REDCap (Vanderbilt University, TN, USA) electronic data capture tools hosted at the UMCG [19, 20].

### 2.2 Participants

Healthy adolescents aged 12–16 years undergoing orthodontic treatment with buccal fixed appliances in both jaws (Metal Twin Brackets, American Orthodontics, WI, USA) for a minimum of 3 months were eligible. Inclusion criteria specified the presence of all permanent anterior teeth from canine to canine in both jaws. Exclusion criteria included multiple participants from the same family/household, allergies to toothpaste components, visits to another dental professional during the intervention period, or syndromic or craniofacial anomalies.

### 2.3 Interventions and Procedures

Eligible participants were approached during their scheduled appointments at the orthodontic postgraduate clinic of the UMCG (timepoint T00). They received information on the study and informed consent, outlining study objectives, procedures and details about the intervention and control toothpastes and their expected effects. Oral hygiene instructions, aligned with the Dutch prevention guidelines and including recommended brushing techniques for at least 2 min twice a day [21], as well as guidance on cleaning fixed appliances and dietary choices, were verbally reiterated and demonstrated to all potential participants by one of the two researchers (SD, LK) during these appointments [21]. Additionally, participants received new manual toothbrushes (TePe GOOD Compact soft, TePe Oral Hygiene Products Ltd., Malmö, Sweden) or electric toothbrush attachments for either Oral‐B (Universal for Oral‐B Soft Bristles, Vardaan Products, Gwalior, India) or Philips (Philips Sonicare ProResults HX6018/07, Amsterdam, the Netherlands) for standardisation purposes.

Potential participants were contacted 1 week after T00 to obtain consent for study participation. Since all patients were between 12 and 16 years old (per the inclusion criteria) Both the participant and one of their parents/guardians were required to co‐sign the consent form. Subsequently, participants were stratified based on their toothbrush habits and randomly allocated to either the intervention or control group. Participants were instructed to avoid using any other toothpaste throughout the study. At their subsequent orthodontic check‐up (T0), the intervention group received the blinded tube containing Mara Expert Plaque Checker toothpaste (Mara Expert Plaque Checker, Health & Beauty International, Tönisvorst, Germany), formulated with blue CI 42090 and red CI 45410 dyes. Meanwhile, the control group received a blinded tube of similarly coloured toothpaste, Colgate Fresh Gel (Colgate‐Palmolive, New York, USA). Participants were advised about the potential effects and appropriate use of the toothpaste and instructed to dispense approximately 0.5 cm of paste onto their toothbrush and distribute it evenly across their teeth before brushing. This preparatory application, conducted without water, was to be maintained for 10 s prior to brushing. Participants were also advised to adopt the new brushing routine immediately after T0 and to continue using the assigned toothpaste until T1.

### 2.4 Outcomes

The primary outcome was the difference in the average plaque fluorescence measured in QLF images taken intraorally from the left, central and right canine‐to‐canine region using the ∆R30 threshold [22, 23]. This difference was measured between images taken at the start (T0) and the follow‐up (T1) during the next orthodontic check‐up. The secondary outcomes assess participants' subjective perceptions through a self‐reported questionnaire administered at T1. Additionally, potential influences of participant characteristics on the primary outcome were examined.

Plaque quantity was assessed at T0 and T1 using QLF images (see Section 2.6). The average plaque fluorescence scores from QLF images of the left lateral, central and right lateral anterior regions at both T0 and T1 were calculated. A reduction of at least 10% in the ∆R30 score was considered the minimal change for significant within‐participant comparison.

### 2.5 Baseline Characteristics

Participant characteristics were collected at T0: brushing habits (manual or electric), age (in years), biological sex at birth, additional oral care practices and socioeconomic status. Additional oral care practices were divided into ‘Yes’ for participants who reported using methods such as interdental cleaning tools or additional fluoride and ‘No’ for those not using any extra oral care methods. Socioeconomic status was stratified into low, medium and high categories based on the level of education attained by participants' mothers: low—mothers who had education up to the primary school level, medium—mothers with preparatory secondary vocational education and high—mothers who had attained bachelor's or master's degrees or higher. Additionally, dental health was assessed using the WHO decayed, missing and filled teeth (DMFT) index.

### 2.6 Study Instruments, Assessor Qualifications and Outcome Data Quality

**FIGURE 1:**
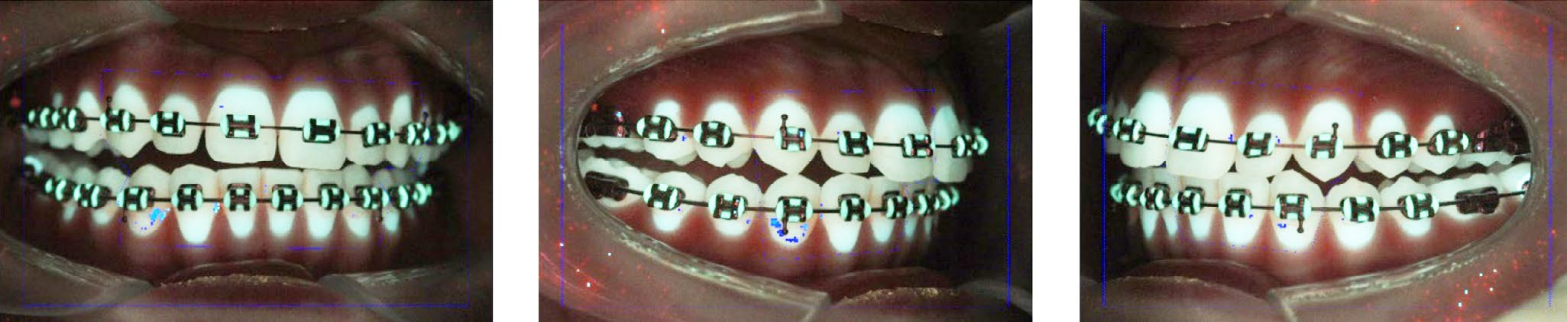
Representative QLF images demonstrating fluorescence thresholding. The blue pixels highlight areas surpassing the ∆R30 fluorescence threshold. The fine dotted blue line indicates the region of interest from canine to canine in the anterior region.

QLF images were captured and analysed using the Pro QLF‐D Biluminator 2 camera and C4 software program (Inspektor Research Systems B.V., Bussum, the Netherlands). The camera settings are provided in Table S1, and cheek retractors were used during capture. Three images (canine‐to‐canine left, central, right) were taken with the teeth 2 mm out of maximal occlusion. All photos were captured in a completely darkened environment (example in Figure 1). The two photographers had been calibrated by Inspektor staff before the start of the trial. During the study, the quality of the QLF images was randomly and anonymously checked by the researchers (SD, LK) to ensure consistent quality. The average percentage of the tooth surface was calculated from canine to canine on the lateral left, central and lateral right images that demonstrated red fluorescence, representing bacterial metabolism in plaque, which exceeded 30% (value ∆R30) thresholds above that of plaque‐free surfaces [22, 23]. A higher ∆R value indicated an area of more active bacterial metabolism in the plaque [22, 23].

A digital questionnaire was developed to address potential concerns related to the toothpaste. This questionnaire underwent a pilot phase with similar patients, representative of the target population who were not eligible for this study, aiming to improve readability, consistency and representativeness. A total of nine questions investigated the participants' brushing experience, oral health improvement, continued use and issues related to the colour, efficacy and taste of the toothpaste. At T1, participants self‐administered this questionnaire to provide feedback on their experiences with the provided toothpaste in the treatment room, immediately after their orthodontic check‐up in the REDCap database. Responses were recorded using a 7‐point Likert scale (1 = *strongly agree*, 2 = *agree*, 3 = *somewhat agree*, 4 = *neutral*, 5 = *somewhat disagree*, 6 = *disagree*, 7 = *strongly disagree*).

### 2.7 Sample Size Determination

A sample size calculation was performed using G*power 3.1 [24]. The calculation was based on an approximate effect size of 10% [17], with an alpha level of 0.05 and a power of 0.8. It was estimated that a total of 33 participants per group would be sufficient to demonstrate a significant change in the average plaque fluorescence score.

### 2.8 Randomisation and Allocation Concealment

Manual brushing and electric brushing habits were treated as distinct participant groups for stratified randomisation. Participants were instructed to maintain their chosen brushing habit for the duration of the trial period. This approach ensured that any potential differences in brushing habits did not influence the outcome and were evenly distributed between the control and intervention groups.

A computer‐generated randomisation sequence was created by the independent researcher not otherwise involved in the study. The randomisation sequence was concealed from the researchers responsible for participant enrolment. Block randomisation was employed, with each block consisting of six participants, ensuring equal allocation between the intervention and control groups. To maintain allocation concealment, sequentially numbered, opaque, sealed envelopes were prepared, each containing the participant's group allocation using a random two‐digit number corresponding to a concealed and blinded toothpaste tube. The envelopes were stored securely, and accessible only to authorised personnel involved in the randomisation process. Prior to assigning interventions, the envelopes were opened sequentially by the researchers responsible for participant enrolment.

### 2.9 Blinding

Participants, caregivers and researchers were blinded for the assignment of interventions. The toothpaste and tubes were designed to be similar in appearance and packaging. All individuals involved, except for the independent researcher responsible for blinding the study, were blinded to the toothpaste type. Participants were asked not to brush their teeth in the clinic for concealment purposes.

### 2.10 Statistical Methods

A single linear regression analysis was performed to analyse the difference in the average plaque fluorescence measured in QLF images using the ∆R30 threshold between the intervention and control groups, adjusted for potential confounding variables. Additionally, a linear univariate regression analysis was conducted to investigate participant‐related factors predicting a reduction in plaque amount. Cronbach's alpha was calculated to assess the internal consistency of the questionnaire responses. Further analyses, such as subgroup and adjusted analyses, were conducted to assess the consistency of the intervention effect across subgroups. The responses to the questionnaire were analysed using the Mann–Whitney *U* test. All statistical analyses were conducted using IBM SPSS Statistics for Windows, version 28.0 (IBM Corp., NY, USA).

## 3 Results

### 3.1 Participant Flow

**FIGURE 2:**
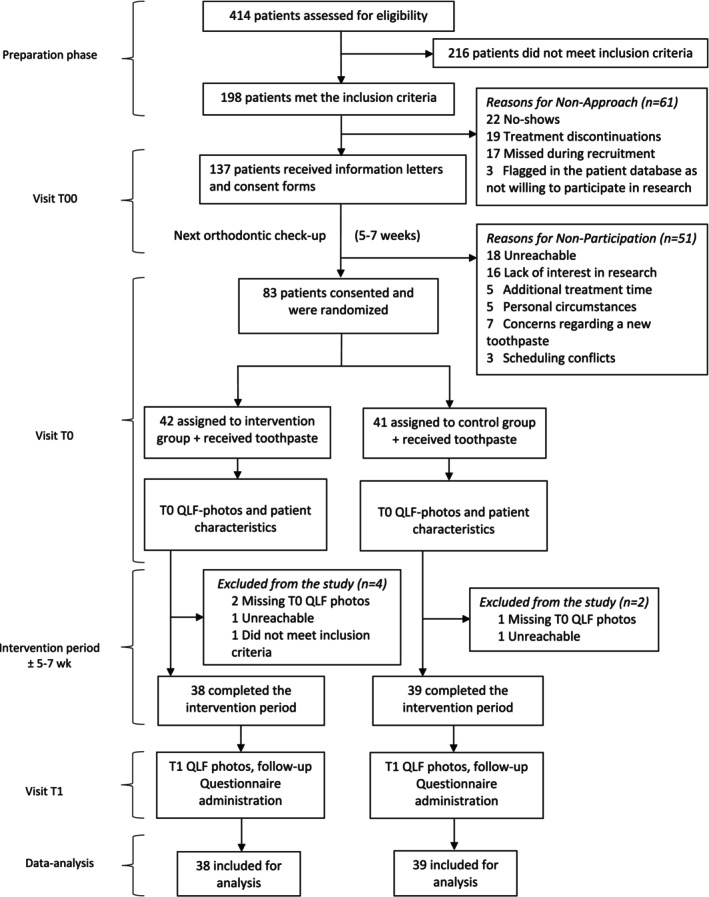
Consort diagram of the study.

The recruitment phase of the study was from October 2021 to January 2022, with the total study duration extending until April 2022. A total of 137 eligible individuals were approached, of whom 83 provided informed consent to participate. During the study, six participants withdrew, resulting in a final cohort of 77 participants, with 38 individuals assigned to the plaque‐identifying group and 39 to the control group (Figure 2). The mean intervention period was 40.22 (SD = 12.01) days.

### 3.2 Baseline Data

The baseline demographic and clinical characteristics for each group are presented in Table 1. The sample comprised 37 manual and 40 electric brushers. The mean age of the participants at T0 was 13.68 (SD = 0.94) years, with the majority being female (67.5%). Approximately half of the participants reported using additional oral care aids (54.5%). Socioeconomic status distribution was low (31.2%), medium (48.1%) and high (20.8%), with similar distributions in the intervention and control groups. The median DMFT score was 0.00 (0–6; IQR = 2.00) for both the intervention and control. The control group had a mean DMFT score of 1 (SD 1.72) and the intervention group had a mean DMFT score of 2 (SD 1.92), with a combined score of 1 (SD 1.82) for both groups.

**TABLE 1:** Baseline characteristics *N* (%) for both study groups.

Variables	Total *n* (%) (*n* = 77)	Intervention group *n* (%) (*n* = 38)	Control group *n* (%) (*n* = 39)
Brushing behaviour (stratified)			
Manual	37 (48.1%)	18 (47.4%)	19 (48.7%)
Electric	40 (51.9%)	20 (52.6%)	20 (51.3%)
Age, years^a^	13.68 (0.94)	13.71 (0.90)	13.64 (0.99)
Sex			
Male	25 (32.5%)	15 (39.5%)	10 (25.6%)
Female	52 (67.5%)	23 (60.5%)	29 (74.4%)
Additional oral care			
Yes	42 (54.5%)	19 (50.0%)	23 (59.0%)
No	35 (45.5%)	19 (50.0%)	16 (41.0%)
Socioeconomic status			
Low	24 (31.2%)	12 (31.6%)	12 (30.8%)
Medium	37 (48.1%)	19 (50.0%)	18 (46.2%)
High	16 (20.8%)	7 (18.4%)	9 (23.1%)
DMFT score^b^	[0: 6.00] (median = 0; IQR = 2.00)	[0: 6.00] (median = 0.50; IQR = 3.25)	[0: 6.00] (median = 0; IQR = 2.00)

Abbreviations: IQR = interquartile range, SD = standard deviation.

^a^ For normally distributed continuous variables, the mean (SD) is given.

^b^ For non‐normally distributed continuous variables, [minimum: maximum] and (median with IQR) are given.

### 3.3 Primary Outcome

The change in average plaque fluorescence score (∆R30) between the plaque‐identifying toothpaste and control groups did not yield statistically significant differences (*F* = 0.211, *p* = 0.647).

Table 2 shows plaque fluorescence scores; the changes are reported as a non‐normally distributed continuous variable, with the median and interquartile range (IQR) presented.

**TABLE 2:** Plaque fluorescence scores (∆R30%) for the intervention and control groups at T0 and T1, and increments (T1–T0).

Variables	Intervention group (*n* = 38)	Control group (*n* = 39)
Plaque score T0^a^	[0: 20.67] (median 5.33; IQR 4.42)	[0: 24.33] (median 4.00; IQR 5.67)
Plaque score T1^a^	[0: 25.00] (median 5.00; IQR 5.17)	[0: 22.33] (median 4.00; IQR 6.33)
Difference in plaque score (T1–T0)	[−13.33: 11.00] (median −0.67; IQR 5.42)	[−16.67: 9.00] (median 0; IQR 3.33)
Reduction of plaque score *N* (%)	22 (57.9%)	16 (41.0%)
Increase of plaque score *N* (%)	15 (39.5%)	16 (41.0%)
Stabilisation of plaque score *N* (%)	1 (2.6%)	7 (17.9%)

^a^ Plaque fluorescence scores, calculated automatically as the percentage of pixels classified as plaque relative to the total buccal crown area (∆R30%) [22]. The ∆R30 values were used for all subsequent analyses and are presented as [minimum: maximum] and (median; interquartile range [IQR]) to show the range and central tendency of the data.

### 3.4 Secondary Outcomes

Table 3 presents the outcomes of participants' experiences at T1. The first four questions pertained to the participants' brushing experience, while the subsequent five questions focused on oral health improvement, continued use and issues related to the colour, efficacy and taste of the toothpaste. Cronbach's alpha coefficient analysis, yielding a value of 0.719, indicated satisfactory internal consistency for the questionnaire.

**TABLE 3:** Paticipants' experience with the provided toothpaste at T1 using a 7‐point Likert scale response.

Questions	Intervention group^a^ (*n* = 38)	Control group^a^ (*n* = 39)	*p* (Sig.)
1. I feel like I'm brushing more consciously with the new toothpaste	[3:6] (median = 5; IQR = 2)	[1:7] (median = 5; IQR = 1)	0.070
2. I feel like I like brushing more with the new toothpaste	[1:7] (median = 4; IQR = 1)	[1:7] (median = 4; IQR = 2)	0.857
3. I feel like brushing is easier with the new toothpaste	[1:7] (median = 4; IQR = 1)	[1:7] (median = 4; IQR = 2)	0.183
4. I feel like I'm brushing away more plaque with the new toothpaste	[2:7] (median = 5; IQR = 1.75)	[1:7] (median = 4; IQR = 1)	0.018*
5. I feel like my oral health has improved with the new toothpaste	[2:7] (median = 5; IQR = 1)	[1:7] (median = 4; IQR = 1)	0.093
6. I would like to continue using this toothpaste	[1:7] (median = 5; IQR = 2)	[1:7] (median = 5; IQR = 2)	0.561
7. I have had no trouble with the colour of this toothpaste	[1:7] (median = 6; IQR = 2)	[1:7] (median = 7; IQR = 1)	0.028*
8. I have had no trouble with the effect of this toothpaste	[3:7] (median = 7; IQR = 1)	[1:7] (median = 7; IQR = 1)	0.329
9. I have had no trouble with the taste of this toothpaste	[1:7] (median = 7; IQR = 3)	[1:7] (median = 7; IQR = 1)	0.101

*Note:* Differences between the two groups were rated on a 7‐point Likert scale (1 = strongly agree, 2 = agree, 3 = somewhat agree, 4 = neutral, 5 = somewhat disagree, 6 = disagree and 7 = strongly disagree).

^a^ The [minimum: maximum] and (median with interquartile range [IQR]) are given.

* *p* < 0.05.

Participants using the plaque‐identifying toothpaste perceived it as more effective for plaque removal (*p* = 0.018), with a median of 5 (IQR: 1.75), compared to the control group's median score of 4 (IQR: 1). Intervention group participants encountered issues with the toothpaste colour, scoring a median of 6 (IQR = 2); the control group reported a median of 7 (IQR = 1), a statistically significant difference (*p* = 0.028). For all other questions, no significant differences existed between the groups.

### 3.5 Additional Analyses

Additional exploratory regression analyses were conducted, incorporating the type of toothpaste as a predictor (Table S2), along with variables such as sex, brushing habits, additional oral care, socioeconomic status, amount of plaque at T0 and DMFT score. None of these variables exhibited a statistically significant effect on the changes in plaque fluorescence score measured by QLF. Further analyses were conducted by categorising the amount of plaque at T0 and the DMFT score into low and high categories around the mean. None of these predictors yielded statistically significant results.

### 3.6 Harms

No harms or unintended effects were reported or observed.

## 4 Discussion

The primary objective of this study was to evaluate the efficacy of a plaque‐identifying toothpaste in adolescents undergoing orthodontic treatment with fixed appliances, in comparison to a regular toothpaste. The present study did not show any significant reduction in plaque fluorescence between the two groups. Results from the participants' experience questionnaire mirrored these findings, with only one item showing a slight advantage towards the plaque‐identifying toothpaste (participant's impression of efficacy in removing plaque) and one against (concerns regarding the colour).

To ensure the reliability of the findings, a blinded randomised controlled trial was designed, utilising a comparator toothpaste with similar colour and composition. Additionally, all participants received oral health instructions based on established guidelines at the outset of the study (T00). However, instructions were given to minimise variation in oral health practices among participants [21]. This methodological rigour provides a comprehensive evaluation of the plaque‐identifying toothpaste's efficacy relative to recognised oral health practices, enhancing our understanding of its potential role in improving oral hygiene.

While the study did not reveal statistically significant differences in plaque reduction, the perceptual benefits noted by participants using the plaque‐identifying toothpaste might have clinical implications in terms of enhancing patient engagement. However, it is possible that these perceptions could lead some patients to overestimate the toothpaste's efficacy, potentially undermining their oral hygiene practices. Consequently, the added value of plaque‐identifying colour in routine brushing appears to be minimal for this population.

Nonetheless, the stronger perception of plaque removal associated with the plaque‐identifying toothpaste may encourage greater adherence to oral hygiene practices, which is particularly important for adolescents undergoing orthodontic treatment. Therefore, although the direct efficacy in reducing plaque accumulation was limited, the motivational impact of the plaque‐identifying toothpaste could indirectly contribute to improved oral health outcomes. For certain patients, especially those facing challenges with adherence, the use of plaque‐identifying toothpaste may serve as a valuable behavioural aid, promoting better overall oral hygiene practices.

This is the first study into the efficacy of a plaque‐identifying toothpaste in adolescents undergoing orthodontic treatment. Previous studies investigating the efficacy of plaque‐identifying toothpaste in other patient groups have utilised various methods to assess the efficacy, such as visual plaque scales or counting bacterial colony growth [16, 17, 18].

Unlike traditional plaque scoring methods that rely on subjective assessments of the amount of plaque, the present study employed QLF to quantify plaque levels, offering several advantages in detecting cariogenic plaque without causing discomfort to patients. QLF offers objective, quantitative measurements for more precise and reproducible data. It detects subtle plaque variations, making it an advanced and reliable tool for clinical and research use [22, 23, 25].

By instructing participants to adhere to established oral health guidelines and employing block randomisation based on brushing habits, confounding factors were minimised [21, 26]. Despite these efforts, the study encountered challenges related to baseline variability in plaque fluorescence scores within the study cohort, although the follow‐up visits were scheduled at consistent times of the day to minimise variability between appointments. Further analyses, which categorised participants based on sex, brushing habits, additional oral care, socioeconomic status, baseline plaque levels (T0) and DMFT score, did not identify statistically significant predictive factors. The variability highlights the intricate nature of assessing plaque and its diurnal fluctuations.

Interestingly, while the group using the plaque‐identifying toothpaste reported a stronger perception of plaque removal, concerns regarding toothpaste colour were noted. However, both groups reported high scores on these items. The lack of significant differences in plaque reduction between the two groups contrasts with previous research demonstrating the efficacy of plaque‐identifying toothpaste in reducing plaque levels in individuals without orthodontic appliances [16]. Factors such as participant age, intervention duration and plaque quantification methods may contribute to these discrepancies.

### 4.1 Limitations

The study has several limitations that may have influenced its findings. Selection bias, as discussed earlier, may have resulted from recruitment challenges, leading to the inclusion of participants with potentially higher motivation for oral hygiene than the general population. This could have introduced confounding variables. Additionally, the short intervention period might not have allowed the full effects to be seen, and the potential presence of the Hawthorne effect—where participants alter their behaviour due to awareness of being observed—could have affected the results [27].

QLF imaging was used to assess outcomes at baseline (T0) and post‐intervention (T1), requiring paired image comparisons for accurate measurement of fluorescence changes. This requirement complicates the use of intention‐to‐treat analyses, as missing follow‐up images would render the primary outcome unmeasurable for those participants. Imputation of missing data from the second time point would compromise the validity of the fluorescence change measurement, as QLF images provide unique, time‐sensitive data that cannot be reliably estimated. Therefore, a per‐protocol analysis was conducted, including only compliant participants with complete paired data, ensuring the most accurate and scientifically sound evaluation of the intervention's efficacy. Standardised oral hygiene instructions were provided to all participants, ensuring consistent conditions, though their influence on the results cannot be ruled out.

### 4.2 Clinical Implications

Whilst the study did not demonstrate statistically significant differences in plaque fluorescence, both groups exhibited an increased awareness of oral hygiene practices. For clinicians, this indicates that plaque‐identifying toothpaste may offer value in promoting patient engagement in oral hygiene, particularly in adolescents at high risk of caries. However, given the minimal impact on actual plaque reduction, regular toothpaste may suffice for most patients and plaque‐identifying toothpaste could be reserved for those who require additional motivation. Further research is warranted to explore the efficacy of integrating plaque‐identifying toothpaste into oral hygiene routines, particularly in high caries risk populations [16]. To better understand the potential benefits of plaque‐identifying toothpaste, such research should focus on long‐term usage and its combination with other oral hygiene techniques. This approach could provide valuable insights into the broader effectiveness of such products in maintaining oral health.

## 5 Conclusions

In the context of the present study, no differences in plaque fluorescence between a plaque‐identifying toothpaste and a regular toothpaste group were found. Participants using the plaque‐identifying toothpaste exhibited a slightly increased awareness of plaque removal. Whilst the plaque‐identifying toothpaste may offer some perceptual benefits for adolescents undergoing fixed appliance therapy, its efficacy in additional plaque reduction is limited.

## Author Contributions

**Sebastiaan P. van Doornik:** conceptualisation, methodology, formal analysis, investigation, resource allocation, original draft writing, review and editing, supervision, project management. **Lisa van Kammen:** conceptualisation, methodology, formal analysis, investigation, resource allocation, original draft writing, project management. **Yijin Ren:** conceptualisation, review and editing. **David J. Manton:** conceptualisation, methodology, review and editing, supervision. **Anne Marie Kuijpers‐Jagtman:** conceptualisation, methodology, review and editing, supervision.

## Disclosure

The study protocol was registered in the ISRCTN registry (number ISRCTN53131830 and can be accessed online at https://www.isrctn.com/ISRCTN53131830).

## Ethics Statement

The Medical Ethics Review Board of the University Medical Center Groningen (UMCG) reviewed the research protocol and determined that the study did not meet the criteria of the Medical Research Involving Human Subjects Act (WMO; registration number: METc 2021/516 RR202100264).

## Conflicts of Interest

The authors declare no conflicts of interest.

## Supporting information


**Table S1.** Camera settings for the Pro QLF‐D Biluminator 2 camera.

**Table S2.** Results of the regression analysis for subgroup analyses, with the type of toothpaste as a predictor, comparing QLF‐plaque score changes between T0 and T1.

Data S1.

## Data Availability

The dataset supporting the findings of this study is accessible online and is provided as a Tables S1 and S2 and Supporting Information S1.
