# Supplementary material for: Effect of a Plaque‐Identifying Toothpaste on Plaque Amount in 12–16‐Year‐Olds With Fixed Orthodontic Appliances: A Randomised, Double‐Blind, Controlled Clinical Trial
Source: Orthod Craniofac Res. 2025 Feb 28;28(4):627–35. doi: 10.1111/ocr.12904 (PMC12233054; doi:10.1111/ocr.12904)
Supplement: Supplementary file 1 — Table S1. Camera settings for the Pro QLF‐D Biluminator 2 camera. [file OCR-28-627-s002.docx]

**Supplementary Table 1.** Camera settings for the Pro QLF-D Biluminator™ 2 camera.

| QLF-D Biluminator | White light | QLF |
| --- | --- | --- |
| Image size | Middle Fine (3456 x 2304) | Middle Fine (3456 x 2304) |
| LED intensity | 100 | 100 |
| ISO speed | ISO 1600 | ISO 3200 |
| Shutter speed | 1/60 s | 1/25 s |
| Aperture | 10.0 | 10.0 |
| White balance | Manual | Daylight |
| Shooting mode | Manual | Manual |
