# Supplementary material for: Effect of a Plaque‐Identifying Toothpaste on Plaque Amount in 12–16‐Year‐Olds With Fixed Orthodontic Appliances: A Randomised, Double‐Blind, Controlled Clinical Trial
Source: Orthod Craniofac Res. 2025 Feb 28;28(4):627–35. doi: 10.1111/ocr.12904 (PMC12233054; doi:10.1111/ocr.12904)
Supplement: Supplementary file 2 — Table S2. Results of the regression analysis for subgroup analyses, with the type of toothpaste as a predictor, comparing QLF‐plaque score changes between T0 and T1. [file OCR-28-627-s001.docx]

**Supplementary Table 2** Regression analysis for subgroups, with the type of toothpaste as predictor, comparing QLF-plaque score changes between T0 and T1.

| Subgroups (Total n = 77) | Constant | Regression coefficient B | R² | 95% CI  *[Low limit ; high limit]* |
| --- | --- | --- | --- | --- |
| Sex | | | | |
| *Male (n = 25)* | 1.200 | -3.267 | 0.089 | [-6.969 ; 0.436] |
| *Female (n = 52)* | 0.080 | 1,122 | 0.018 | [-1.218 ; 3.463] |
| Brushing behavior |  | | | |
| *Manual (n = 37)* | -0.351 | -0.057 | 0.000 | [-3.524 ; 3.411] |
| *Electric (n = 40)* | 1.050 | -0.850 | 0.016 | [-3.059 ; 1.359] |
| Additional oral care |  | | | |
| *Yes (n = 42)* | -0.306 | 0.102 | 0.000 | [-3.031 ; 3.235] |
| *No (n = 35)* | 1.444 | -1.428 | 0.042 | [-3.853 ; 0.998] |
| Socioeconomic status |  | | | |
| *Low (n = 24)* | 0.528 | -2.722 | 0.088 | [-6.608 ; 1.163] |
| *Medium (n = 37)* | -0.333 | 1.579 | 0.031 | [-1.426 ; 4.584] |
| *High (n = 16)* | 1.556 | -1.651 | 0.072 | [-5.046 ; 1.745] |
| Amount of plaque at T0 |  | | | |
| *Low (n = 39)* | 1.275 | -0.650 | 0.012 | [-2.586 ; 1.285] |
| *High (n = 38)* | -0.938 | 0.331 | 0.001 | [-3.251 ; 3.914] |
| DMFT score |  | | | |
| *Low (n = 40)* | 1.317 | -0.177 | 0.001 | [-2.628 ; 2.274] |
| *High (n = 37)* | -0.741 | -0.575 | 0.004 | [-3.698 ; 2.547] |
